# Supplementary material for: Reduction of stillbirth rate in refugee and migrant populations living on the Thailand Myanmar border: A retrospective study 1986–2023
Source: PLOS Glob Public Health. 2026 May 12;6(5):e0005983. doi: 10.1371/journal.pgph.0005983 (PMC13166957; doi:10.1371/journal.pgph.0005983)
Supplement: S1 Appendix — (DOCX) [file pgph.0005983.s001.docx]

Table A: Missing values or not available as introduced after 1986.

| **value n, (%)** | **Data collection** | **Liveborn**  **N=64,380** | **Stillborn**  **N=721** |
| --- | --- | --- | --- |
| Maternal age |  | 29 (<1%) | 1 (<1%) |
| Parity |  | 7 (<1%) | 4 (<1%) |
| Anaemic |  | 6,861 (10.7%) | 120 (16.6%) |
| Smoking | Data collection commence in 1997 | 11,183 (17.4%) | 192 (26.6%) |
| ANC visit in first trimester |  | 0 (0%) | 0 (0%) |
| First trimester BMI | Height collection commenced in 2003 | 45,032 (69.9%) | 613 (85.0%) |
| Literacy | Data collected commenced standardly in 2008 | 33,049 (51.3%) | 443 (61.4%) |
| Refugee, Migrant |  | 0 (0%) | 0 (0%) |
| Number of ANC visits |  | 6 *(<1%)* | 2 *(<1%)* |
| Malaria in pregnancy |  | 0 (0%) | 0 (0%) |
| Syphilis | Screening commenced in 2012 | 40,939 (63.6%) | 521 (72.3%) |
| HIV | Screening commenced in 2008 | 38,243 (59.4%) | 500 (69.3%) |
| Antepartum Haemorrhage |  | 9,394 (14.6%) | 139 (19.3%) |
| Hypertension in pregnancy |  | 0 (0%) | 0 (0%) |
| Pre-eclampsia |  | 0 (0%) | 0 (0%) |
| Eclampsia |  | 0 (0%) | 0 (0%) |
| EGA at birth |  | 0 (0%) | 0 (0%) |
| Congenital abnormality |  | 573 (0.9%) | 90 (12.5%) |
| Birthweight |  | 8,572 (13.3%) | 266 (36.9%) |
| Place of birth |  | 5,907 (9.2%) | 107 (14.8%) |

Table B: Definition of causes of stillbirth

| Causes | Definition |
| --- | --- |
| APH | Vaginal bleeding during or before birth, from EGA≥28 |
| Congenital abnormality | Any abnormality observed based on systematic physical exam, severe enough for causing stillbirth. |
| Maternal Infection | Fever within 72 hours before or during labour. |
| Placenta insufficiency | Small for gestational age based on birthweight. Intra Uterine Growth restriction based on ultrasound, reversed or absent umbilical flow, Small fundal height for gestational age. At pathology any signs of abnormal placenta. |
| Obstructed labour/prolonged labour | Obstructed labour, Prolonged labour, shoulder dystocia, other presentation then vertex with prolonged labour/obstructed labour. |
| Hypertensive disease | Hypertension in pregnancy, Pre-Eclampsia, Eclampsia |
| Cord | Cord prolapse, constricting cord around the neck |
| Trauma | Trauma resulting from a fall, uterine massage or pressure by a traditional birth attendant, with a following report of loss of foetal movement |
| Uterine rupture | Uterine rupture |
| Maternal Illness | Systemic maternal illness, as SLE or Diabetes. |
| Acute Intrapartum moment | Any acute moment during second stage of labour after which there was no fetal heartbeat. |

Table C: Classification of the stillbirths according the ICD 10 code amongst confirmed antepartum and intrapartum cases.

|  | **Maternal medical condition** | | | | |  |
| --- | --- | --- | --- | --- | --- | --- |
|  | **M1 Complications of placenta, cord and membranes** | **M2 Maternal complications of pregnancy** | **M3 Other complications of labor and delivery** | **M4 Maternal medical and surgical conditions** | **M5 No maternal condition/not specified** | **Causes Total (%)** |
| Causes of antepartum deaths |  | | | | | |
| A1: Congenital malformations, deformations and chromosomal abnormalities | 2 | 0 | 0 | 5 | 29 | 36 (6.3%) |
| A2: Infection | 11 | 0 | 0 | 7 | 0 | 18 (3.1%) |
| A3: Antepartum hypoxia | 140 | 0 | 0 | 44 | 3 | 187 (32.6%) |
| A4: Other specified antepartum disorder | 1 | 0 | 0 | 0 | 1 | 2 (0.3%) |
| A5: Disorders related to foetal growth | 4 | 0 | 2 | 5 | 24 | 35 (6.1%) |
| A6: Foetal death of unspecified cause | 4 | 0 | 0 | 25 | 119 | 148 (25.8%) |
| Causes of intrapartum deaths |  | | | | | |
| I1: Congenital malformations, deformations and chromosomal abnormalities | 0 | 3 | 4 | 0 | 7 | 14 (2.4%) |
| I2: Birth trauma | 1 | 0 | 8 | 0 | 3 | 12 (2.1%) |
| I3: Acute intrapartum event | 37 | 0 | 58 | 6 | 5 | 106 (18.5%) |
| I4: Infection | 2 | 0 | 0 | 1 | 0 | 3 (0.5%) |
| I5: Other specified intrapartum disorder | 0 | 0 | 0 | 0 | 0 | 0 (0%) |
| I6: Disorders related to foetal growth | 0 | 0 | 2 | 0 | 3 | 5 (0.9%) |
| I7: Intrapartum death of unspecified cause | 0 | 2 | 1 | 1 | 4 | 8 (1.4%) |
| Maternal condition total (%) | 202 (35.2%) | 5 (0.9%) | 75 (13.1%) | 94 (16.4%) | 198 (34.4%) | 574 (100%) |

Table D: Characteristics antepartum and intrapartum stillbirth.

|  |  | **Livebirth** *(n=64,380 )* | **Antepartum Stillbirth**  *(n=425)* | **Intrapartum Stillbirth**  (n=149) |
| --- | --- | --- | --- | --- |
| Mean^ Age |  | *26.1 (6.6) [13-53]* | *29.3 (7.6) [15-45]* | 27.3 (7.0) [16-45] |
| Age group | *< 18 years*  *18-34 years*  *≥ 35 years* | *6.1 (3,929/64,351)*  *80.3 (51,670/64,351)*  *13.6 (8,752/64,351)* | *4.0 (17/425)*  *66.4 (282/425)*  *29.6 (126/425)* | 5.4 (8/149)  75.8 (113/149)  18.8 (28/149) |
| Mean^ Parity |  | *2 (2) [0-17]* | *3 (3) [0-12]* | 2 (2) [0-8] |
| Parity group | *0*  *1-4*  *>4* | *33.1 (21,249/64,273)*  *57.6 (36,997/64,273)*  *9.4 (6,027/64,273)* | *25.0 [106/424]*  *52.4 [222/424]*  *22.6 [96/424]* | 39.6 (59/149)  47.0 (70/149)  13.4 (20/149) |
| Anaemic |  | 35.1 (20,165/57,519) | 43.1 (170/394) | 44.7 (59/132) |
| Smoking |  | *19.4* (42,902/53,197) | *31.7 (114/360)* | 28.0 (33/118) |
| ANC visit in 1st trimester |  | *42.6 (27,448/64,380)* | *38.4 (163/425)* | 36.2 (54/149) |
| Mean^ 1st trimester BMI |  | *21.2 (3.2) [13.5-45.1]* | *21.3 (3.4) [15.7-33.2]* | 21.7 (4.0) (16.6-34.3) |
| Asian BMI group *kg/m^2^* | *<18.5  ≥18.5- <23*  *≥23- <25*  *≥25* | *17.3 (3,350/19,348)*  *59.3 (11,479/19,348)*  *11.9 (2,297/19,348)*  *11.5 (2,222/19,348)* | *17.6 (19/108)*  *57.4 (63/108)*  *12.0 (13/108)*  *13.0 (14/108)* | 14.7 (5/34)  61.8 (21/34)  2.9 (1/34)  20.6 (7/34) |
| Literacy |  | 65.2 (20,437/31,331) | 58.9 (113/192) | 46.8 (29/62) |
| Status  Migrant | Refugee  Migrant | 55.0 (35,407/64,380)  45.0 (28,973/64,380) | 58.6 (249/425)  41.4 (176/425) | 57.7 (86/149)  42.3 (86/149) |
| Number of ANC visits | ≥4  ≥8 | 88.2 (56,762/64,374)  69.4 (44,675/64,374) | 75.8 (322/425)  51.8 (220/425) | 74.5 (111/149)  56.4 (84/149) |
| Malaria in pregnancy | *P. vivax*  *P. falciparum* | 5.9 (3,777/64,380)  6.8 (4,394/64,380) | 4.2 (18/425)  12.2 (52/425) | 4.0 (6/149)  10.7 (16/149) |
| Syphilis |  | 0.6 (137/23,441) | 1.4 (2/142) | 0 (0/43) |
| HIV |  | 0.5 (118/26,137) | 2.0 (3/151) | 2.0 (1/51) |
| Antepartum Haemorrhage |  | *0.7 (391/54,986)* | 20.5 (79/385) | 12.0 (15/125) |
| Hypertension in pregnancy |  | *5.0 (3,206/64,380)* | 9.9 (42/425) | 8.7 (13/149) |
| Pre-eclampsia |  | 1.8 (1,129/64,380) | 6.4 (27/425) | 2.7 (4/149) |
| Eclampsia |  | *0.2 (149/64,380)* | 0 (0/425) | 2.0 (3/149) |
| Mean^ EGA birth, weeks |  | *39 (1.8) (28-45]* | 34 (4) [28-44] | 37 (4) [28-43] |
| Preterm birth group |  | *0.8 (514/64,380)*  *7.9 (5,062/64,380)*  *91.3 (58,804/64,380)* | 28.0 (119/425)  35.8 (152/425)  36.2 (154/425) | 12.1 (18/149)  22.8 (34/149)  65.1 (97/149) |
| Congenital abnormality |  | 1.5 (966/63,807) | 14.5 (57/393) | 15.7 (21/134) |
| Small for gestational age** |  | *23.4 (13,032/55,808)* | 47.5 (151/318) | 40.4 (40/99) |
| Large for gestational age** |  | *3.0 (1,696/55,808)* | 2.2 (7/318) | 7.1 (7/99) |
| Mean^ Birthweight g |  | *2,954 (468) [600-5580]* | *1,872 (779) [520-3820]* | 2,527 (800) [600-4100] |
| Place of birth | SMRU  Home  Hospital  Other | *66.1 (38,658/58,473)*  *23.2 (13,573/58,473)*  9.5 (5,544/58,473)  *1.2 (698/58,473)* | *65.8 (263/400)*  *5.5 (22/400)*  *28.0 (112/400)*  *0.8 (3/400)* | 42.6 (58/136]  16.2 [22/136]  39.7 [54/136]  1.5 [2/136] |
| Caesarean section |  | *4.6 (2,964/64,380)* | *NA* | 15.4 (23/149) |
| Type of vaginal birth | Vertex  Breech  Other  Instrumental | *96.8 (59,448/61,416)*  *1.3 (807/61,416)*  *0.2 (101/61,416)*  *1.8 (1060/61,416)* | *NA* | 62.7 (79/126)  23.0 (29/126)  1.6 (2/126)  12.6 (16/126) |

^Data are % (n/N) unless otherwise stated, Abbreviations: e.g. ANC antenatal care, EGA estimated gestational age, g grams, N.A. not applicable,^

^^Mean values consistently expressed as^ *^Mean (sd) [min-max]^*

^*reference <4 ANC for ANC ≥4 visits and <8 for ANC ≥8 visits; ** reference normal weight^
