# Supplementary material for: Reduction of stillbirth rate in refugee and migrant populations living on the Thailand Myanmar border: A retrospective study 1986–2023
Source: PLOS Glob Public Health. 2026 May 12;6(5):e0005983. doi: 10.1371/journal.pgph.0005983 (PMC13166957; doi:10.1371/journal.pgph.0005983)
Supplement: Attachment — Submitted filename: Review of the manuscript of Taco Jan Prins & Collaborators.docx [file pgph.0005983.s004.docx]

1. **Summary**

It was an honour for us to appear among the ones who had to review manuscript titled : « *Reduction of stillbirth rate in refugee and migrant populations living on the Thailand Myanmar border: a retrospective study 1986-2023 »* written by Taco Jan Prins and collaborators from Chiang Mai University, which cover a period of 37 years as shown in the tittle. After a deep reading we noticed that the main idea was to identify the causes and circonstances of stillbirth during the prenancy and the birth in  this kind of marginalized population and what was the possible paths for reducing the issue. The authors noticed that the main causes were the blooding during the pregnancy betwen 28 weeks of estimated age and the birth, congenital malformation and the maternal infections(specially the malaria) and other specifical ones followed in there frequency as collected in interval of 4 years in the period of study. Beyond that were social condition of desplaced people (refugees and migrants) without health assurance. The good news from the study was inspite of marginalisation and lack of health assurance of refugees and migrants, cases of stillbirth decreased in the decades according the assistance for access to antenatal care services for this people in need.The used method to collect data and setting study are very clear,well shown and current statistics measures used to analyze it.

2. **Discussion of specific ares to improvement.**

I didn’t notice major issue. I think that they can only improve the perspective for next study. Specially, the relashionship between stress of migration.

3. **Other points**

No comment here.
