# Supplementary material for: Reduction of stillbirth rate in refugee and migrant populations living on the Thailand Myanmar border: A retrospective study 1986–2023
Source: PLOS Glob Public Health. 2026 May 12;6(5):e0005983. doi: 10.1371/journal.pgph.0005983 (PMC13166957; doi:10.1371/journal.pgph.0005983)
Supplement: Attachment — Submitted filename: Response to Reviewers.docx [file pgph.0005983.s007.docx]

PGPH-D-26-00283
Reduction of stillbirth rate in refugee and migrant populations living on the Thailand Myanmar border: a retrospective study 1986-2023
PLOS Global Public Health

Dear Dr. Taco Jan Prins,

Thank you for submitting your manuscript to PLOS Global Public Health. After careful consideration, we feel that it has merit but does not fully meet PLOS Global Public Health’s publication criteria as it currently stands. Therefore, we invite you to submit a new version with minor changes of the manuscript that addresses the points raised during the review process. If any suggested revisions cannot be accommodated, please explain the reasons for their exclusion.

Please submit your revised manuscript by April 3, 2026. If you will need more time than this to complete your revisions, please reply to this message or contact the journal office at globalpubhealth@plos.org. When you're ready to submit your revision, log on to [https://www.editorialmanager.com/pgph/](https://url.uk.m.mimecastprotect.com/s/EGxfCO5D2f2o62sEfLSGtL_9?domain=editorialmanager.com) and select the 'Submissions Needing Revision' folder to locate your manuscript file.

Please include the following items when submitting your revised manuscript:

- A letter that responds to each point raised by the editor and reviewer(s) should be prepared and uploaded as a separate file labeled *“Response to Reviewers.”* If any suggested revisions cannot be accommodated, please provide a clear explanation for the reasons behind their exclusion.
- A marked-up copy of your manuscript that highlights changes made to the original version. You should upload this as a separate file labeled 'Revised Manuscript with Track Changes'.
- An unmarked version of your revised paper without tracked changes. You should upload this as a separate file labeled 'Manuscript'.

We look forward to receiving your revised manuscript.

Kind regards,

Natia Skhvitaridze, M.D. MBA., Ph.D
Academic Editor
PLOS Global Public Health

Additional Editor Comments:

**Please** review your reference list to ensure that it is complete and correct. If you have cited papers that have been retracted, please include the rationale for doing so in the manuscript text, or remove these references and replace them with relevant current references. Any changes to the reference list should be mentioned in the rebuttal letter that accompanies your revised manuscript. If you need to cite a retracted article, indicate the article’s retracted status in the References list and also include a citation and full reference for the retraction notice.

Reviewers' comments:

**Comments to the Author**

1. Does this manuscript meet PLOS Global Public Health’s [publication criteria](https://url.uk.m.mimecastprotect.com/s/z7DSCP5E9f5q75H0hESxHItA?domain=track.editorialmanager.com)? Is the manuscript technically sound, and do the data support the conclusions? The manuscript must describe methodologically and ethically rigorous research with conclusions that are appropriately drawn based on the data presented.

Reviewer #1: Yes

Reviewer #2: Yes

2. Has the statistical analysis been performed appropriately and rigorously?

Reviewer #1: Yes

Reviewer #2: Yes

3. Have the authors made all data underlying the findings in their manuscript fully available (please refer to the Data Availability Statement at the start of the manuscript PDF file)?

The [PLOS Data policy](https://url.uk.m.mimecastprotect.com/s/1ynbCQ5G2fNm5NhMigSGEiHR?domain=track.editorialmanager.com) requires authors to make all data underlying the findings described in their manuscript fully available without restriction, with rare exception. The data should be provided as part of the manuscript or its supporting information, or deposited to a public repository. For example, in addition to summary statistics, the data points behind means, medians and variance measures should be available. If there are restrictions on publicly sharing data—e.g. participant privacy or use of data from a third party—those must be specified.

Reviewer #1: Yes

Reviewer #2: Yes

4. Is the manuscript presented in an intelligible fashion and written in standard English?

PLOS Global Public Health does not copyedit accepted manuscripts, so the language in submitted articles must be clear, correct, and unambiguous. Any typographical or grammatical errors should be corrected at revision, so please note any specific errors here.

Reviewer #1: Yes

Reviewer #2: Yes

5. Review Comments to the Author

Please use the space provided to explain your answers to the questions above. You may also include additional comments for the author, including concerns about dual publication, research ethics, or publication ethics.

Reviewer #1:

1. Summary
It was an honour for us to appear among the ones who had to review manuscript titled: « Reduction of stillbirth rate in refugee and migrant populations living on the Thailand Myanmar border: a retrospective study 1986-2023 » written by Taco Jan Prins and collaborators from Chiang Mai University, which cover a period of 37 years as shown in the tittle. After a deep reading we noticed that the main idea was to identify the causes and circumstances of stillbirth during the pregnancy and the birth in this kind of marginalized population and what was the possible paths for reducing the issue. The authors noticed that the main causes were the blooding during the pregnancy between 28 weeks of estimated age and the birth, congenital malformation and the maternal infections(specially the malaria) and other specifical ones followed in there frequency as collected in interval of 4 years in the period of study. Beyond that were social condition of displaced people (refugees and migrants) without health assurance. The good news from the study was in spite of marginalisation and lack of health assurance of refugees and migrants, cases of stillbirth decreased in the decades according the assistance for access to antenatal care services for this people in need. The used method to collect data and setting study are very clear, well shown and current statistics measures used to analyze it.

Thank you for the comment.

2. Discussion of specific areas to improvement.
I didn’t notice major issue. I think that they can only improve the perspective for next study. Specially, the relationship between stress of migration.

Thank you for the comment, and we are working on tools to measure stress accurately in this mixed language populations

3. Other points
No comment here.

Reviewer #2:

This is an important study that quantifies the progress and persistent challenges in reducing stillbirths for limited studied marginalized population (refugee and migrant women). Expressing sincere appreciation for the authors for conducting this research. The findings have direct implications for policy and practice in similar humanitarian and low-resource settings.

I find it suitable for publication after a minor revision to address the following points and comments:
- Line 52: the death rate mentioned 16.2 for South Asia, this rate covers both central and South Asia (this should be precisely mentioned or alternatively refer to the 16.7 for South Asia only, reference to the same report “Never forgotten”.

Thank you for the comment. It is changed to 16.7.
- Line 53: Europe SBR is 3.0 as per the same report mentioned in the previous point.

Thank you for the comment, it is changed to 3.0
- Line 60: suggest clarification on and addressing the continuum of care in this point of quality care.

Thank you this has been addressed and changed

*In theory, most stillbirths are preventable if care is accessible and of quality across the continuum of care, including timely and appropriate antenatal, intrapartum and referral care*

- Line 85: has this electronic shift in all clinics at the same time? Please clarify or explain differences.

This has been changed to

*The clinical details were extracted from medical records which were initially paper based and became electronic from 2008, with one clinic at a time making the transition, as direct mentorship was required.*

*And:*

*The population and clinics in the period of time have been described in more detail, including maps, in the SMRU Refugee and Migrant Pregnancy Cohort [14] .*

- Lines 96 - 97: mentioning either side of the borders while it is mentioned this clinic is located in the Thailand side of the border line (are you referring to IDPs (internally displaced population, be specific)), revise this and make it clear for readers. Suggest adding some background on the main dynamics of the two sides of the border in the introduction or under the setting section of the methods.

Thank you this has been added, see the next comment

- Lines 97 – 100: are the SMRUs in Myanmar or Thailand side of the border, these lines are a bit confusing (please review and ensure clarify how the setup of SMRUs system is at its current status and level from which data was collected/extracted!

Thank you this has been changed and added:

*Since 1986, the SMRU has built up a system of clinics providing ANC and perinatal care on both sides the Thai-Myanmar border in Tak Province Thailand. The initial focus besides perinatal care was screening and treating of malaria, which causes high morbidity among this population. Services were developed first for refugee women in camps in Thailand (between 1986 and 2016) followed by services for migrant women (from either side of the border). Migrant clinics provided ANC starting from 1997 and from 2007 perinatal care. After closure of SMRU’s last refugee clinic in 2016 and later with the COVID-19 pandemic, clinics on the Myanmar side of the border were opened in 2017 and 2020 to serve the marginalized and displaced populations – who can be labelled as internally displaced [17]. The migrant population in Thailand and internally displaced population inside Myanmar fluctuate, for example decreasing with the semi-democratic rule in the late 2010s and increasing with the armed conflict of the 2021 coup d’etat. The population and clinics in the period of time have been described in more detail, including maps, in the SMRU Refugee and Migrant Pregnancy Cohort [14].*

- Line 101: In brief, specify the “other ethnic groups” for clarity, neutrality & credit.

Thank you this has been added:

*other ethnic groups as Shan or Mon*
- Line 106: It will be valuable to know, how many sites covered in the study and how many records, explain the number of facilities and types (clinics, hospitals) and locations (Thailand/Myanmar??) from which the records were collected by type of records (paper/electronic)

We agree that some details is useful here. Rather than put a map we have referred the reader to reference 14 where this detail and maps are provided. We have differentiated clinics as SMRU facilities and made it clear that C-sections are referred to hospitals, in the paragraph on settings.

Thailand and Myanmar have used the same data collection (paper/electronic) but we have not emphasized this (and do not wish to).

*SMRU has no access to the Thai or Myanmar Government hospitals and collect the data retrospectively after discharge.* This has been added.

*The population and clinics in the period of time have been described in more detail, including maps, in the SMRU Refugee and Migrant Pregnancy Cohort [14].*

- Lines 153 -156: Revisit and clarify this sentence: Due to unknown timing of death most stillbirths at home had an unknown timing of the stillbirth, unless there was a strong indication for antepartum (e.g. severe growth restriction or maceration) or intrapartum (arrived in the clinic with head entrapment at breech delivery).

Thank you, this sentence has been changed:

*Due to unknown timing of death (eg antepartum or intrapartum) most stillbirths at home had an unknown timing of the stillbirth, unless there was a strong indication for the timing of death, for antepartum (eg. severe growth restriction or maceration) or intrapartum (arrived in the clinic with head entrapment at breech delivery, or prolonged entrapment was well described by patient/patient relative/traditional birth attendant and noted in the record).*

- Line 212: Results (Table 1): The presentation of percentages is slightly confusing, suggest reviewing and remove the repetition of total number in “Causes N=721 (%)”.

Thank you this has been removed.
- Line 228: Table 2 page 13: there are missing data for all variables in the table, suggest adding a footnote to mention this for clarity, for example in the Place of birth the denominator is 58,473, but the total livebirths is 64,380 leading to around 5,900 missing their location, was this because this was not recorded (need to clarify this particular variable but also for other variables in the table).

Thank you for the comment: this has already been described:

*Line 127 Missing data per variable extracted is presented in a table (S1 Table ).*

*And has been added in the results again: Missing data per variable extracted is available (S1 Table ).*
- Line 275: the term quality healthcare should be used within the population context (migration and refugee), there researchers have not referenced to the quality standards for healthcare in such context and this is important to correlate to this situation.

Thank you this has been changed:

*Further strengthening the relations between facilities with basic emergency obstetric care [29] and hospital care to improve referral and risk management has the potential to improve the outcomes further.*
- Lines 284 – 286: support this statement by a reference.

Reference has been added. :

*and hospital care to improve referral and risk management has the potential to improve the outcomes further[30].*

- Lines 292-296: the authors refer to interesting suggestions on occupational hazards, to strengthen this point, could they add a reference to a study documenting the occupational hazards (e.g., agricultural work in heat) or housing conditions of this specific population, if such data exists?

Thank you the reference has been added:

*IOM. Overview of Myanmar Migrants in Thailand Food Education Rent Repaying debt Remittances Health [Internet]. 2025 [cited 2026 Mar 12]. Report. Available from:* [*https://thailand.iom.int/sites/g/files/tmzbdl1371/files/documents/2025-03/myanmar_migrants_thailand_jan25_final-1.pdf*](https://thailand.iom.int/sites/g/files/tmzbdl1371/files/documents/2025-03/myanmar_migrants_thailand_jan25_final-1.pdf)

Thank you and we agree it would be great to have occupation but this history is not routinely available – we are trying to examine the question of heat in a separate analysis but the relatively low number of stillbirths is unlikely to be helpful. There is a great proportion of Preterm Birth and Small for Gestational age infants that will be examined.

- Line 305: the authors used “Anomaly” here, suggest using the same terminology across the different sections for consistency, although I would recommend using anomaly/anomalies rather than abnormality/abnormalities, but I leave this to authors decision.

Thank you for the useful comment, as we also described in earlier publications it as abnormality we will use this here again. However appreciate the suggestion to change it into anomaly.

- Lines 312-313: revise the sentence for clarity and consider rephrasing to “limit the ability to assign an exact cause …”

Thank you this has been changed.

- Lines 339-341: please support this statement by reference(s).

Reference has been added: Wungrath J. Antenatal Care Services for Migrant Workers in Northern Thailand: Challenges, Initiatives, and Recommendations for Improvement. Journal of Child Science. Georg Thieme Verlag; 2023. p. E118–26. doi:10.1055/s-0043-1772844

- Lines 343-345: is this a finding from this study if so, make it clearer or please support this statement by reference(s).

Thank you this has been changed to:

*These collaborations should be strengthened to increase the access to healthcare for migrants.[50] Another report shows that these collaboration could be complicated by financial support, lack of communication or unequal partnerships and of course, the capacity to absorb care of marginalised populations [48].*
- Lines 463 – 465 Reference #18 and Line 540-542 Reference #43 seems to be identical and need consolidation, please review and correct.

Thank you these are indeed the same and are made into one.

Additional comments:

1. Discussion of the Plateau 2010-2023, the observation that the stillbirth rate has plateaued since approximately 2010, despite increased hospital births, is a critical finding. The discussion touches on this (lines 277-286, 343-345) but could be strengthened by a more in-depth exploration of potential reasons. Could this be due to the harder-to-reach women who have more complex risk factors? Limitations in the quality of care within referral hospitals (e.g., delays in emergency C-sections, different clinical protocols)? Persistent barriers at the "third delay" at facility, the impact of increasing political instability in Myanmar, especially post-2021, on the health and stress levels of the population? Expanding on these possibilities would add significant value to the discussion.

Thank you this has been added:

*This may be partly explained by delays occurring at different levels of care, including presentation to the first health facility (which may be exacerbated by crisis such as COVID-19 and armed conflict), referral processes and delays in receiving timely caesarean section at the referral hospital.*

2. In brief discussion of similar context and findings from other humanitarian settings and conflict affected countries covering similar marginalized groups would be great additions and strengthen the manuscript.

This had been added:

*The findings of this study are supported by from other conflict-affected settings. Similar challenges have been described among marginalized and displaced populations in settings such as Lebanon and Gaza, where conflict and limited access to maternal health services and contribute to adverse perinatal outcomes.[50,51] Armed conflict disrupts health systems, results in displacement, reducing the quality of living conditions and shelter, increasing food insecurity and psychosocial stress among pregnant women. These factors, together with barriers to accessing antenatal and obstetric care, have been associated with adverse pregnancy outcomes, including low birthweight and preterm birth.[52]*

3. Missing Data and impact on findings: The issue of missing data is acknowledged (line 122, S1 Table) and in the footnotes of Table 3. While the authors have handled this by noting adjustments, the potential for bias remains. Could the authors comment briefly in the discussion on the potential direction of bias this might introduce? For example, is it likely that women with missing literacy data are more or less vulnerable? Discussion of this limitation would strengthen the manuscript.

Thank you, the topic of the literacy was already been touched in the Alinea before:

*Literacy was associated with a lower risk of intrapartum stillbirth. As previously reported in this population during the COVID-19 pandemic, literate women were less associated with loss to follow up [17]. This suggests that literate women are more likely to access to healthcare in childbirth compared to illiterate women. This study did not look into health literacy specifically, but as reported earlier, health literacy, understanding of the medical advice and dangers signs given during the antenatal care and perinatal outcomes are closely related [44].*

*How we describe missingness is important. Missingness typically refers to data that was expected to be collected and for a range of reason it is not done, forgotten, misplaced, no recorded i.e. truly missing. We have attempted to distinguish this from data that can’t exist i.e. prior to a certain date it is simply not collected (expected in this prolonged cohort), such as literacy which commenced in 2008 (S1 Table). We don’t think literacy levels would change drastically before and after we started collecting it. As the reviewer noted, we have adjusted for this, in case.*

The following sentences has been added to the discussion:

*Loss to follow‑up may not have occurred at random, as women lost to follow‑up may have been more vulnerable and at higher risk of adverse outcomes, thereby potentially biasing the results. Further population‑based research should aim to capture outcomes across the entire population.*

4. The conclusion "Improving access to health clinics and skilled attendance at birth has the potential to reduce preventable stillbirths" is correct, at the same time the paper also shows that after improving access, the rate plateaued. This might need slightly more elaboration on higher-level health system barriers and harmful community practices.

Thank you the conclusion has been sharpened:

*This analysis provides evidence that stillbirth rates among refugee and migrant women can be reduced, even in the context of intermittent conflict. By increasing access to basic quality care, treating malaria and ensuring skilled birth attendance, preventable stillbirths can be reduced. However, the observed plateau in stillbirth suggests that achieving parity with the host population remains very difficult due to persistent differences in access to timely and adequate care all levels of health systems between refugee, migrant, and host communities.*

In conclusion, this is a well-conducted and important study. The revisions requested are minor and aimed at enhancing the clarity, depth, and impact of the manuscript. The language, while generally clear, contains a number of minor errors that should be corrected before final acceptance. The data availability statement is appropriate for such vulnerable population. I am confident that the authors can address these points, and I recommend the manuscript for acceptance pending these minor revisions.

Thank you

6. PLOS authors have the option to publish the peer review history of their article ([what does this mean?](https://url.uk.m.mimecastprotect.com/s/NWnPCR5J0fg8AgtOs1S1UzHY?domain=track.editorialmanager.com)). If published, this will include your full peer review and any attached files.


**Do you want your identity to be public for this peer review?** If you choose “no”, your identity will remain anonymous but your review may still be made public.


For information about this choice, including consent withdrawal, please see our [Privacy Policy](https://url.uk.m.mimecastprotect.com/s/mXSRCV0jkFP1DPHQtpSEX-HU?domain=track.editorialmanager.com).

Reviewer #1: **Yes:** Roger Paluku Hamuli

Reviewer #2: No

[NOTE: If reviewer comments were submitted as an attachment file, they will be attached to this email and accessible via the submission site. Please log into your account, locate the manuscript record, and check for the action link "View Attachments". If this link does not appear, there are no attachment files.]

Figure Resubmissions:

While revising your submission, we strongly recommend that you use PLOS’s NAAS tool ([https://ngplosjournals.pagemajik.ai/artanalysis](https://url.uk.m.mimecastprotect.com/s/DMuHCWj0lTyvAyCpuNSolpLq?domain=ngplosjournals.pagemajik.ai)) to test your figure files. NAAS can convert your figure files to the TIFF file type and meet basic requirements (such as print size, resolution), or provide you with a report on issues that do not meet our requirements and that NAAS cannot fix.

After uploading your figures to PLOS’s NAAS tool - [https://ngplosjournals.pagemajik.ai/artanalysis](https://url.uk.m.mimecastprotect.com/s/DMuHCWj0lTyvAyCpuNSolpLq?domain=ngplosjournals.pagemajik.ai), NAAS will process the files provided and display the results in the "Uploaded Files" section of the page as the processing is complete. If the uploaded figures meet our requirements (or NAAS is able to fix the files to meet our requirements), the figure will be marked as "fixed" above. If NAAS is unable to fix the files, a red "failed" label will appear above. When NAAS has confirmed that the figure files meet our requirements, please download the file via the download option, and include these NAAS processed figure files when submitting your revised manuscript.

Thank you, Fig1, Fig2 and S2 Fig have been updated according the requirements needed.
